# Critical Illness-Associated Cerebral Microbleed in a Young Patient With Sickle Cell Disease

**DOI:** 10.7759/cureus.86166

**Published:** 2025-06-16

**Authors:** Ruth Pius, Moustafa M Shaaban, Jose A Suarez

**Affiliations:** 1 Internal Medicine, Lincoln Medical Center, Bronx, USA; 2 Internal Medicine, St. George's University, True Blue, GRD

**Keywords:** cerebral microbleed (cmb), invasive mechanical ventilation, medical intensive care unit (micu), sickle cell disease: scd, stroke

## Abstract

Critical illness-associated cerebral microbleeds (CICMs) are a recent clinical entity described as occurring in critically ill and mechanically ventilated patients, especially those with a risk of cerebral hypoxia. CICMs have been associated with progressive cognitive decline, and the management is supportive. We report the case of a 29-year-old male patient with sickle cell disease admitted for vaso-occlusive crises, which was complicated by multifocal pneumonia, and acute chest syndrome requiring intensive care unit admission and mechanical ventilation. He was noted to have poor mental status off sedation, with a brain MRI showing innumerable microhemorrhages throughout the bilateral cerebral and cerebellar hemispheres, suggestive of CICM.

## Introduction

Sickle cell disease patients have a high incidence of cerebrovascular events, such as ischemic and hemorrhagic strokes, such that about 11% of sickle cell patients experience stroke prior to age 20. However, cerebral microbleeds are a rare occurrence in sickle cell disease. [1]. More broadly, critical illness-associated cerebral microbleeds (CICMs) are microhemorrhages that have been recently described in ICU patients who are critically ill; specifically, those with a high risk of cerebral hypoxia, and are mechanically ventilated [2]. Cerebral microbleeds, generally, are defined as the accumulation of small blood products in brain tissue that appear as small circular or elliptical lesions with a size of 2 to 10 mm [3]. It is seen in varying frequencies in different conditions. However, it is very important to detect early due to its potential complications. These microbleeds are often asymptomatic lesions that can be found incidentally and are associated with an increased risk of cognitive decline, dementia, intracerebral hemorrhage, cerebral infarction, or recurrence of transient ischemic attack, and mortality [3,4]. While CICMs have garnered more attention, there is still much to be investigated, such as their pathophysiology, clinical significance, prognosis, and treatment [2,5]. Here, we report the case of a young critically ill patient with sickle cell disease who developed cerebral microbleeds during a difficult ICU course.

## Case presentation

**Figure 1:**
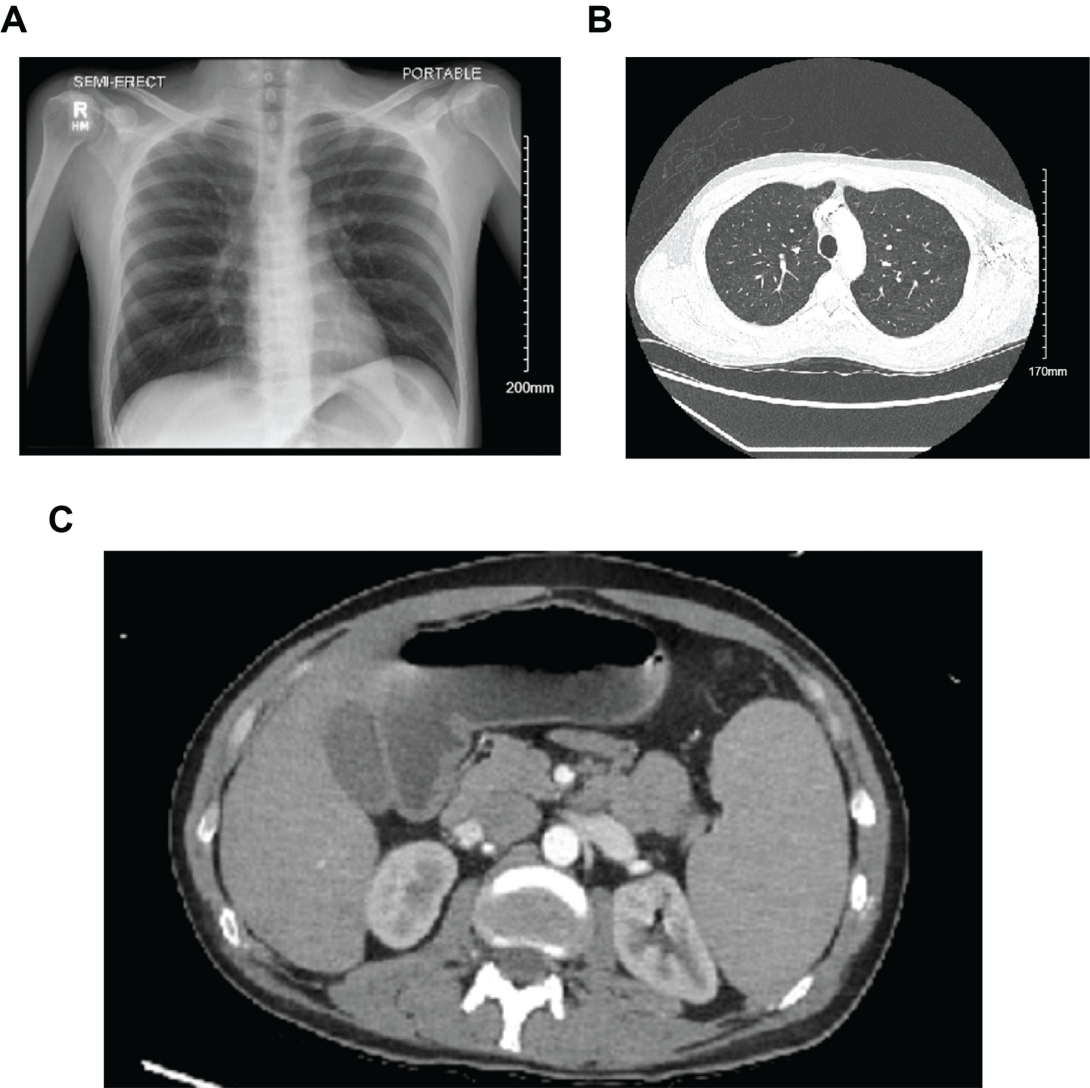
Unremarkable initial chest X-ray-PA view (A), computed tomography (CT) angiogram of the chest-axial view (B), and CT abdomen and pelvis-axial view (C) PA: posteroanterior

A 29-year-old male patient with sickle cell disease and alcohol use disorder presented to the emergency department with chest, back, and upper and lower extremity pain for one day, without fever, chills, or cough. Vital signs were within normal limits, other than a tachycardia of 109 bpm. On physical examination, the patient was found to be in painful distress and to have abdominal tenderness and signs of dehydration. Laboratory findings (Table 1) showed hemoglobin (hb) of 11.9 g/dl (baseline of 8-9 mg/dl), leukocytosis with neutrophil predominance, mild thrombocytopenia, unconjugated hyperbilirubinemia, elevated lactate dehydrogenase, metabolic acidosis, and an initial elevated lactate level. Concerning imaging, the clear X-ray, computed tomography (CT) angiography of the chest, abdomen, and pelvis were unremarkable (Figure 1). 

**Table 1:** Trend of laboratory results during hospitalization

Parameter	Patient values	Reference range/units
Initial labs		
Hemoglobin	11.9 (baseline 8-9)	14-18 g/dl
White blood cells	15.46	4.80-10.8 x 10(3)/mcl
Platelet	137	150-450 x 10(3)/mcl
Lactate dehydrogenase	400	135-225 U/L
Reticulocyte %	5.17	0.50-2.00 %
Albumin	4.8	3.5-5.2 g/dl
Total bilirubin	7.72	0.20-1.20 mg/dl
Direct Bilirubin	2.00	0.00-0.30 mg/dl
Lactate	10.3	0.5-2.2 mmol/l
Venous pH	7.33	7.32-7.43
Creatinine	0.82	0.70-1.20 mg/dl
Blood urea nitrogen	9.0	6.0-23.0 mg/dl
Bicarbonate	18	22-29 mmol/l
Day 2 of admission		
Lactate	1.4	0.5-2.2 mmol/l
Hemoglobin	6.6	14-18 g/dl
Platelet	66	150-450 x 10(3)/mcl
Day 4		
Hemoglobin	9.3	14-18 g/dl
Platelet	28	150-450 x 10(3)/mcl
White blood cell	3.75	4.80-10.8 x 10(3)/mcl
Reticulocyte %	4.87	0.50-2.00%
Lactate dehydrogenase	3600	135-225 U/L
Day 7		
Lactate dehydrogenase	1940	135-225 U/L
Day 13		
Hemoglobin	8.4	14-18 g/dl
Platelet count	226	150-450 x 10(3)/mcl

**Figure 2:**
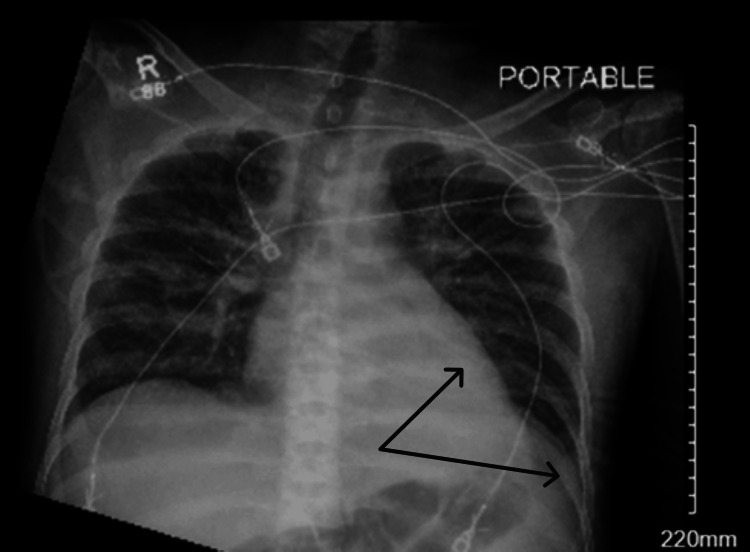
Chest X-ray showing new left lower lobe and retrocardiac opacity

These findings prompted the patient’s admission to the medicine floor for vaso-occlusive crisis, while the lactic acidosis was thought to be due to heavy alcohol use immediately before admission. The next day, the patient began developing a fever, with a body temperature of 38.2℃. A repeat chest X-ray showed a new left lower lung lobe infiltrate (Figure 2), prompting the commencement of antibiotics for pneumonia.

**Figure 3:**
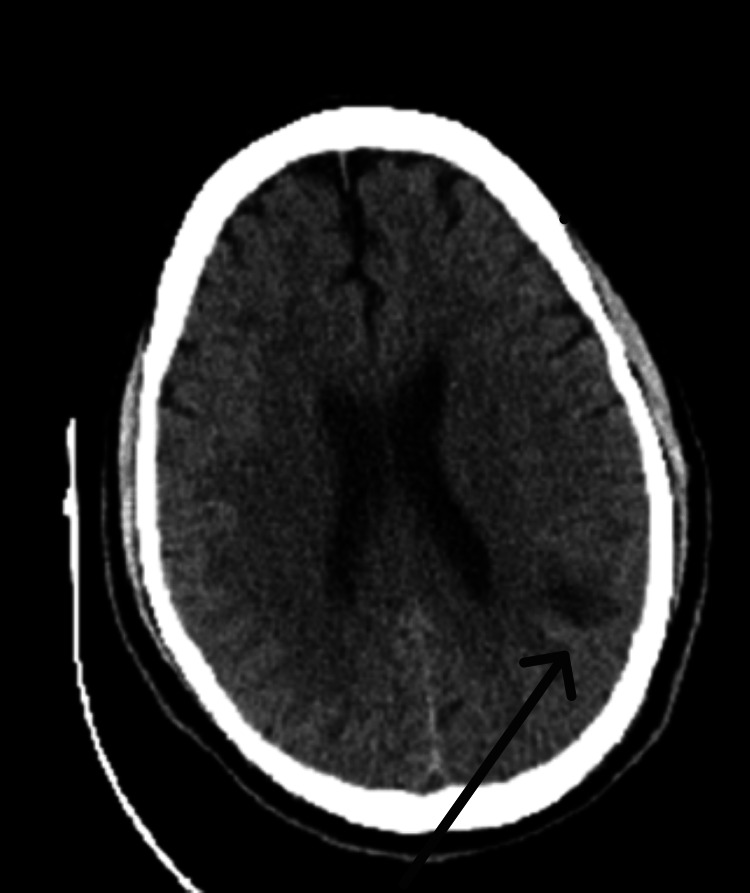
CT head showing ovoid area of hypoattenuation in the left parietal subcortical white matter.

On the following day, he was found to have an oxygen saturation of 87% along with tachycardia (140 bpm) and tachypnea (22 breaths/minute). He was also noted to develop alcohol withdrawal symptoms, which required the administration of lorazepam, after which his mental status deteriorated. Due to his worsening clinical status, the patient was upgraded to the medical intensive care unit (ICU) for suspected acute chest syndrome along with alcohol withdrawal, since he needed closer monitoring and required high-flow nasal oxygen. In the ICU, he quickly had worsening acute hypoxic respiratory failure, as well as further deterioration of his mental status; therefore, he was intubated. A computed tomography scan of the head, which was done to investigate the cause of his worsening mental status, revealed hypoattenuation of left parietal subcortical white matter suggestive of acute stroke (Figure 3).

The patient was also found to have a drop in hemoglobin, which was thought to be due to hemolysis, evidenced by his elevated unconjugated bilirubin and reticulocyte count, and a drop in platelet count (Table 1). The patient's international normalized ratio (INR) was 1.2, activated partial thromboplastin time (APTT) was 25.7, and fibrinogen was 241; therefore, he was thought not to be in disseminated intravascular coagulation (DIC). He received three packed red blood cells to restore his hemoglobin level close to his baseline, thereby reducing sickling.

**Figure 4:**
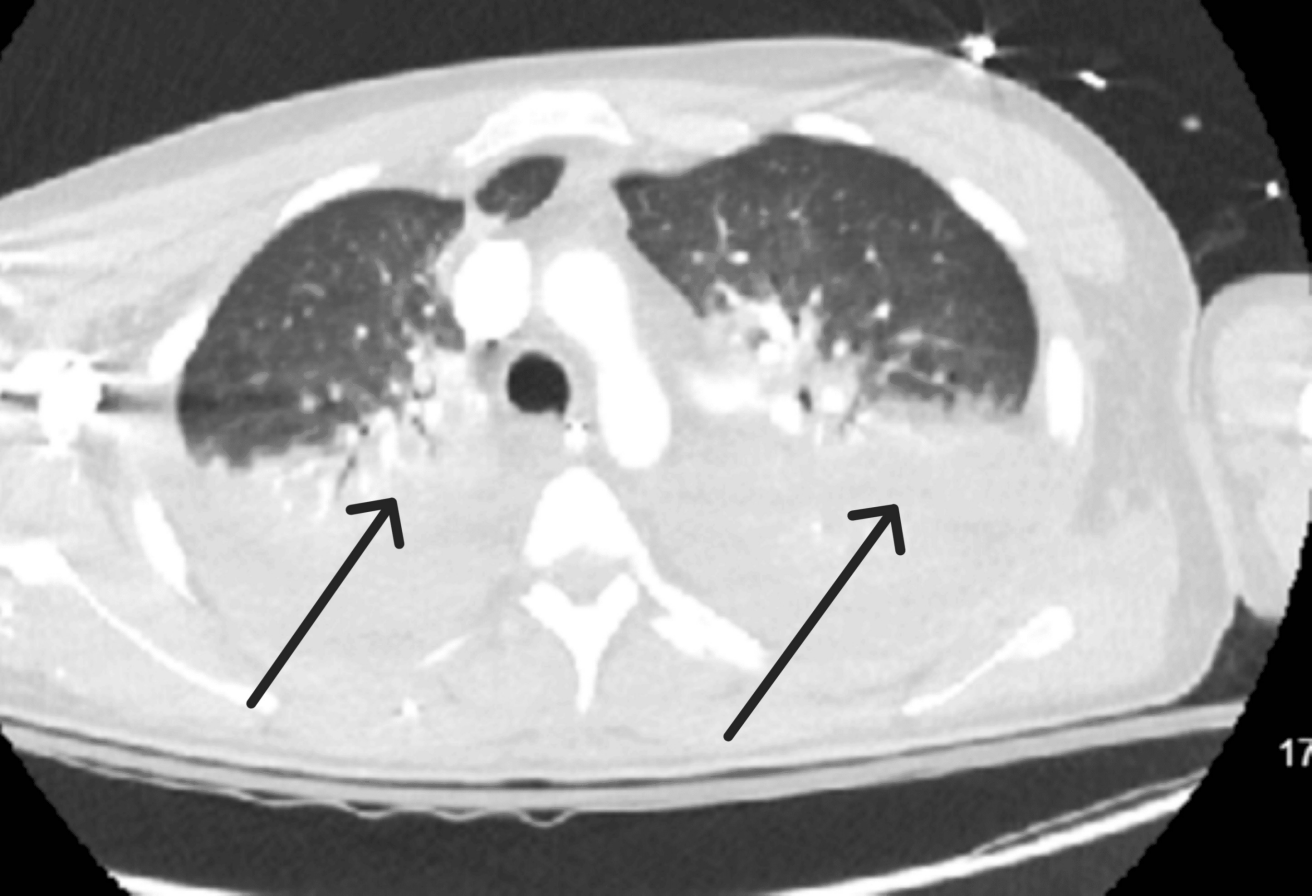
CT chest showing multifocal infiltrates and atelectasis

The patient's clinical status continued to worsen with persistent fever and low blood pressure; therefore, he was treated for septic shock, requiring low-dose vasopressor and continued mechanical ventilation. A chest CT was performed, showing multilobar infiltrates (Figure 4), further raising the concern for acute chest syndrome, thereby necessitating an exchange blood transfusion. 

**Figure 5:**
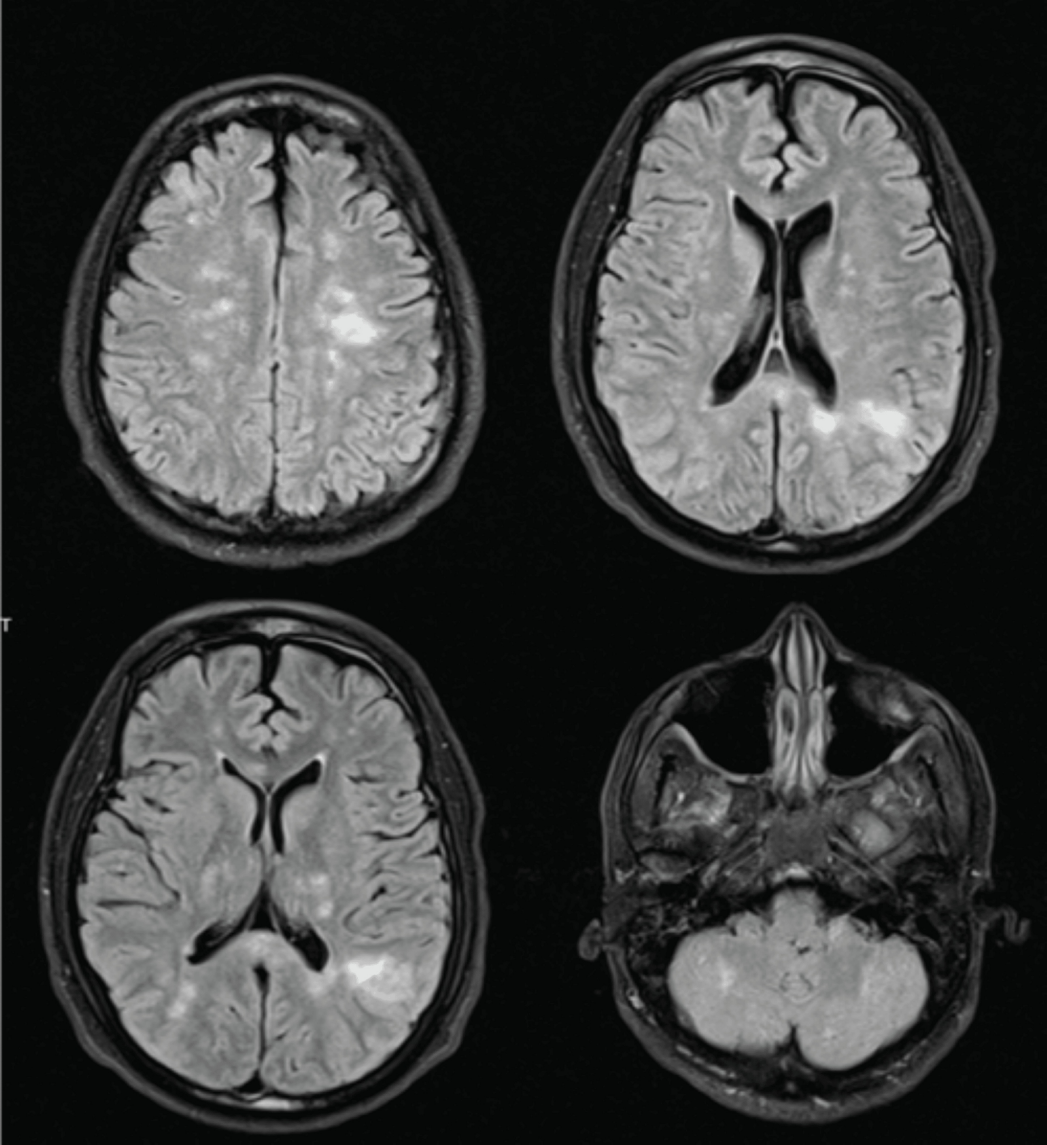
T2-FLAIR magnetic resonance imaging (MRI) showing numerous foci of hypointensity in the cerebral and cerebellar hemispheres consistent with microbleeds. Also showing multiple foci of T2/FLAIR hyperintensity within subcortical white matter and bilateral thalami, corona radiata and centrum semiovale; as well as T2/FLAIR hyperintensity within the cerebellar white matter FLAIR: fluid-attenuated inversion recovery; MRI: magnetic resonance imaging

After the transfusion, his hemoglobin-S level remained below 13.2%. His platelet count subsequently improved to normal within 10 days after the initial drop without platelet transfusion (Table 1), and the drop was thought to be related to sepsis-induced thrombocytopenia as well as heavy alcohol use. When off sedation, the patient exhibited poor mental status as well as a lack of spontaneous limb movement, unexplained by the left parietal stroke, therefore requiring further neurologic investigation. At this time, he was no longer in septic shock and was off pressors. Brain MRI was done (Figure 5), which showed innumerable microhemorrhages throughout the bilateral cerebral and cerebellar hemispheres with multifocal hyperintensities within the cerebral hemispheres, with a background of diffuse T2/fluid-attenuated inversion recovery (FLAIR) hyperintensity and restricted diffusion in the cerebral white matter, suggesting multifocal stroke likely due to the abrupt hypotensive episodes, and microhemorrhages suggestive of critical-Illness associated cerebral microbleeds. 

After failed spontaneous breathing trials and continued need for ventilation, the patient was transitioned to tracheostomy ventilation. While his neurological status improved throughout his admission, it remained far below his baseline. He was able to track faces, follow commands, and respond to simple questions by nodding his head. He was subsequently transferred to a long-term acute care hospital to wean off the ventilator, where he was able to be weaned off after two months. The patient was subsequently moved to a nursing home due to the limitations in their functional status. 

## Discussion

While the topic of cerebral microbleeds in its broader sense is well-studied, the pathophysiology of CICM is not fully understood and much less reported. Cerebral microbleeds are typically visualized with brain MRI and are found in different areas of the brain depending on the cause of the microbleed or the patient’s risk factors. For example, basal ganglia and thalamic microbleeds predominate in hypertension (HTN), while parietal and occipital cortical microbleeds predominate in cerebral amyloid angiopathy [6]. Cerebellar microbleeds, on the other hand, are less reported and have not been found to be associated with specific risk factors or causes [7].

In sickle cell disease, factors such as vaso-occlusive crises, hemolysis, and inflammation promote vasculopathy, which predisposes sickle cell patients to having stroke [1] and may also be implicated in microbleeds. It has been suggested that cerebral microbleeds in patients with sickle cell may be a consequence of fat emboli, which typically cause punctate foci of restricted diffusion in the brain, causing a starfield pattern seen on brain MRI, but this pattern is not seen in CICM [5]. Cerebral microbleeds that are associated with critically ill patients have been found to affect different areas of the brain than other types of cerebral microbleeds. For instance, in HTN, cerebral microhemorrhage lesions are more common in the basal ganglia, pons, cerebellum, and thalami, while microhemorrhages occur mostly in the gray-white matter junction of the cerebral cortex in cerebral amyloid angiopathy [2]. 

CICM has characteristic microbleed patterns that show a predilection for the white matter tracts, sparing the gray matter, with a typical distribution in the subcortical and juxtacortical white matter and corpus callosum. CICMs could also involve the internal capsule, external capsule, and cerebellar peduncles [5,8], as reported in our patient, who had bilateral cerebellar microbleeds. A study of multiple patients with CICM found that the microbleeds consistently appeared in the same region [5]. One study showed CICMs to be more commonly seen in patients requiring acute respiratory distress syndrome (ARDS) rescue therapies, including intubation and mechanical ventilation [9]. Another study also found a link between microhemorrhages and critically ill COVID-19 patients [10]. It is also important to note that patients with high-altitude hypoxia have been found to have corpus callosum predominant microbleeds. The findings of these studies and similarities in imaging across all patients suffering from severe hypoxic status suggest that, despite a lack of full clarity on the mechanism, hypoxemia could be a common factor associated with disruptions in the blood-brain barrier through possible hypoxia-induced hydrostatic or chemical effects, leading to extravasation of erythrocytes or microbleeds [5]. Specifically, sickle cell patients could be at increased risk due to the risks of recurrent vaso-occlusive hypoxemia, if their brain is chronically deprived of oxygen. In our patient, intubation and mechanical ventilation were present as known risk factors, similar to those reported in other cases of CICM.

It is important to be aware of these microbleeds as their occurrence can change the management of stroke, a common comorbid condition in critically ill patients. The use of thrombolytic agents, a common treatment for strokes, should be used with caution in patients with cerebral microbleeds due to the possibility of exacerbation of bleeding events. It has been shown that there is a relatively increased risk of cerebral hemorrhage and mortality in patients with microbleeds, and more symptomatic hemorrhage occurs with a higher number of bleeds [3]. These concerns can alter the management of these patients as well as encourage closer observation. It is also worth investigating other treatments commonly used in critically ill patients that may also worsen or be dangerous to administer in patients who suffer from cerebral microbleeds.

## Conclusions

To conclude, although there are many causes of cerebral microbleeds, CICMs specifically appear to be strongly associated with cerebral hypoxia and mechanical ventilation in the setting of respiratory failure. These microbleeds are found in the juxtacortical white matter and corpus callosum and spare the deep and periventricular white matter and the gray matter. They may also occur in the cerebellum, albeit this is less reported. Cerebral microbleeds often come with many comorbidities as well as increased risk for mortality and are associated with increased risk of cerebral stroke complications and progressive cognitive decline. The early identification of patients with cerebral microbleeds can change management recommendations in settings of acute stroke, and this is of high relevance to high-risk patients, such as sickle cell disease patients. CICM presents several diagnostic challenges due to a lack of standardized criteria and a nonspecific clinical presentation. Further investigations are required to establish treatment and management guidelines, with emphasis on developing standardized imaging follow-up protocols, validating risk stratification tools to identify high-risk patients in order to manage CICM, slow or inhibit the potentially associated cognitive decline and dementia, and improve overall outcomes.
